# Biological Monitoring of Human Exposure to Neonicotinoids Using Urine Samples, and Neonicotinoid Excretion Kinetics

**DOI:** 10.1371/journal.pone.0146335

**Published:** 2016-01-05

**Authors:** Kouji H. Harada, Keiko Tanaka, Hiroko Sakamoto, Mie Imanaka, Tamon Niisoe, Toshiaki Hitomi, Hatasu Kobayashi, Hiroko Okuda, Sumiko Inoue, Koichi Kusakawa, Masayo Oshima, Kiyohiko Watanabe, Makoto Yasojima, Takumi Takasuga, Akio Koizumi

**Affiliations:** 1 Department of Health and Environmental Sciences, Kyoto University Graduate School of Medicine, Kyoto, 6068501, Japan; 2 Department of Food Nutrition, Kyoto Bunkyo Junior College, Uji, 6110041 Japan; 3 Department of Health and Nutrition, Kyoto Koka Women’s University, Kyoto, 6150882, Japan; 4 Shimadzu Techno-Research Incorporated, Nishinokyo, Kyoto, 6048435, Japan; Aichi Cancer Center Research Institute, JAPAN

## Abstract

**Background:**

Neonicotinoids, which are novel pesticides, have entered into usage around the world because they are selectively toxic to arthropods and relatively non-toxic to vertebrates. It has been suggested that several neonicotinoids cause neurodevelopmental toxicity in mammals. The aim was to establish the relationship between oral intake and urinary excretion of neonicotinoids by humans to facilitate biological monitoring, and to estimate dietary neonicotinoid intakes by Japanese adults.

**Methodology/Principal Findings:**

Deuterium-labeled neonicotinoid (acetamiprid, clothianidin, dinotefuran, and imidacloprid) microdoses were orally ingested by nine healthy adults, and 24 h pooled urine samples were collected for 4 consecutive days after dosing. The excretion kinetics were modeled using one- and two-compartment models, then validated in a non-deuterium-labeled neonicotinoid microdose study involving 12 healthy adults. Increased urinary concentrations of labeled neonicotinoids were observed after dosing. Clothianidin was recovered unchanged within 3 days, and most dinotefuran was recovered unchanged within 1 day. Around 10% of the imidacloprid dose was excreted unchanged. Most of the acetamiprid was metabolized to desmethyl-acetamiprid. Spot urine samples from 373 Japanese adults were analyzed for neonicotinoids, and daily intakes were estimated. The estimated average daily intake of these neonicotinoids was 0.53–3.66 μg/day. The highest intake of any of the neonicotinoids in the study population was 64.5 μg/day for dinotefuran, and this was <1% of the acceptable daily intake.

## Introduction

Neonicotinoid pesticides have been widely used to protect vegetables, rice, and fruit trees because they are effective at controlling a range of pests, particularly shield bugs and aphids. The ecological impacts of neonicotinoid pesticides on invertebrates and their predators have recently been causing concern.[1, 2] Seven neonicotinoid pesticides are used in many of the Japanese prefectures because they are not very toxic to humans.[3] However, the European Food Safety Authority (EFSA) reviewed the data available for three neonicotinoid pesticides (clothianidin, imidacloprid, and thiamethoxam) and evaluated their impacts on bees in January 2013. The EU Council imposed regulations on the use of these three pesticides in 2013.[4] The EFSA evaluated the potential developmental and neurological toxicities of acetamiprid and imidacloprid in December 2013.[5]

The Japanese Food Safety Commission estimated that each Japanese adult consumes 1050 μg/d of acetamiprid, 206 μg/d of clothianidin, 713 μg/d of dinotefuran, 307 μg/d of imidacloprid, and 265 μg/d of thiamethoxam.[6–10] However, these estimates were derived from the maximum values found in a pesticide residue study and were made assuming that processing and cooking food does not decrease the residual pesticide concentration. A method for assessing human exposure to neonicotinoid pesticides using actual measurements is urgently required. It is also necessary to identify convenient biomarkers for neonicotinoid exposure so that the biological monitoring method can be fully established.

Imidacloprid, clothianidin, and dinotefuran have been found to be excreted in urine with short biological half-lives in animal experiments,[11, 12] so it is likely that the daily intake of these neonicotinoids could be estimated from their concentrations in urine samples. Neonicotinoid pesticides have been detected in human urine,[13, 14] but the relationship between oral intake and urinary excretion of neonicotinoids in humans has not yet been described.

In this study, the four main neonicotinoid pesticides that are used in Japan (acetamiprid, clothianidin, dinotefuran, and imidacloprid) were studied with the aim of establishing a biological monitoring method. The Japanese production volume of each of these pesticides was more than 50 t in 2012.[3] Human subjects took oral microdoses of the pesticides in a deuterium-labeled neonicotinoid study and a non-deuterium-labeled neonicotinoid study, and urine samples were collected from each participant. The urine samples were analyzed for the neonicotinoids and their possible metabolites. Toxicokinetic modeling was then performed, and the dietary intakes of neonicotinoids by the general Japanese population were evaluated using the concentrations found in urine samples provided by 373 Japanese adults.

## Materials and Methods

### Experimental design and study population

**Fig 1:**
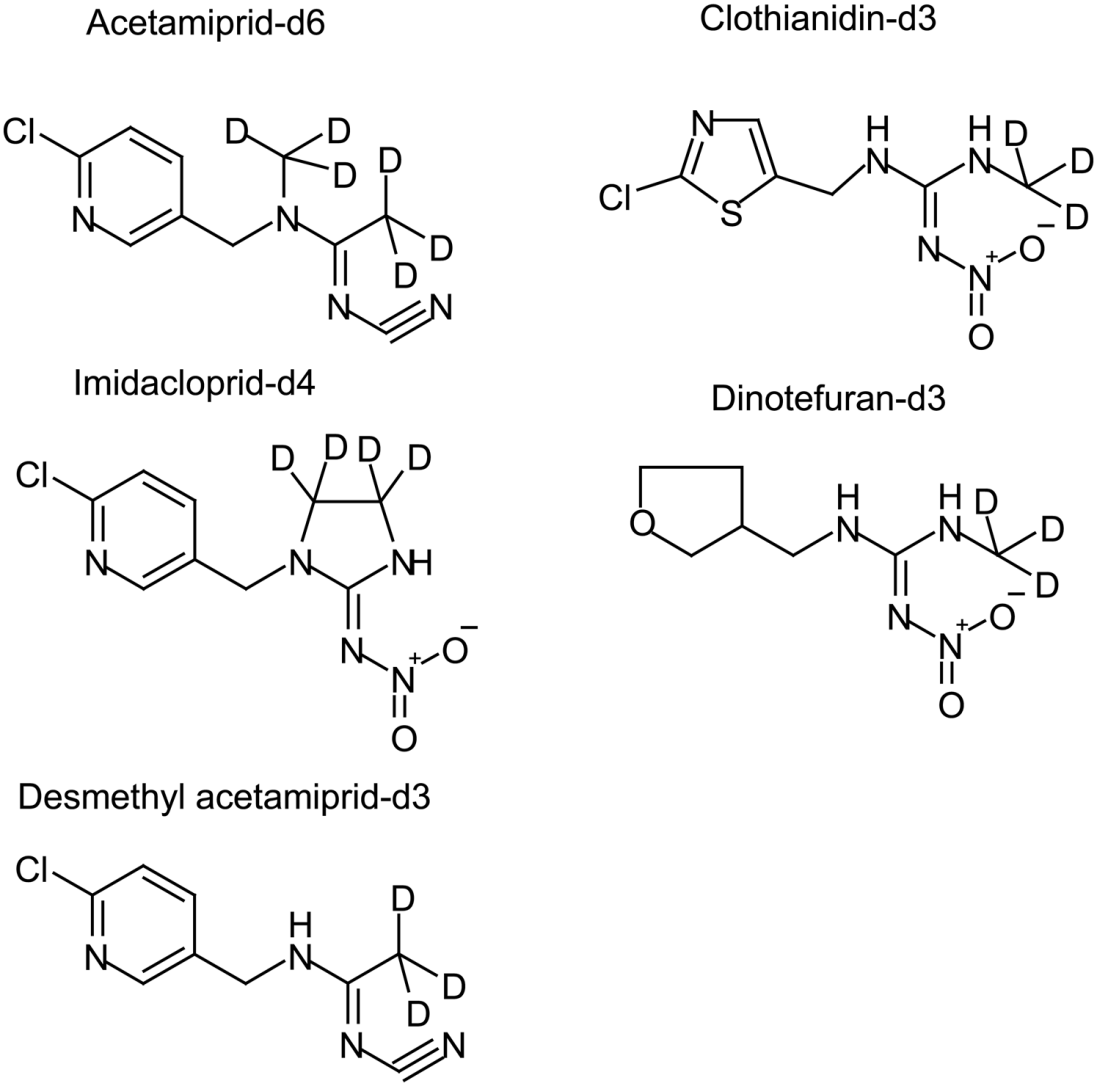
Deuterium-labeled neonicotinoids used in the dosing study.

A single microdose of a mixture of deuterium-labeled neonicotinoids (5 μg each of acetamiprid-d6, clothianidin-d3, dinotefuran-d3, and imidacloprid-d4) (Fig 1) was orally ingested by each of nine healthy adults (S1 Table), and 24 h pooled urine samples were collected from each participant for 4 consecutive days afterwards. A non-deuterium-labeled neonicotinoid microdose study in which each of 12 healthy adults ingested a single oral dose of a mixture of 2 μg each of acetamiprid, clothianidin, dinotefuran, and imidacloprid was then conducted to validate the model derived from the deuterium-labeled microdose study. In the non-labeled study, 24 h pooled urine samples were collected on the day before and the day after the microdose was ingested, then a spot urine sample was collected from each participant every 24 h until 168 h after the dose had been ingested. Every participant in the microdose study gave written informed consent before participating. The research protocol for the microdose study was reviewed and approved by the Kyoto University Graduate School of Medicine ethics committee (approval no. E2166; approval date 10 June 2014).

A survey was also conducted in which a spot urine sample was collected from each of 373 Japanese adults in Uji City and Kyoto City, Japan, between January 2009 and December 2014 (Table 1). Most of the samples were collected in 2014 (n = 293). Each participant completed a self-reported questionnaire in which they gave their age, number of children delivered, smoking history, and consumption of vegetables the day (full 24 h) before their urine was collected, and answered questions on pesticide use in their home. The urine samples were stored at −30°C at the Kyoto University Human Specimen Bank[15] until they were analyzed. Every participant gave written informed consent before participating in the cross-sectional study. The research protocol for the study was reviewed and approved by the Kyoto University Graduate School of Medicine ethics committee (approval nos. E25 and E2166; approval dates 14 November 2003 and 10 June 2014, respectively).

**Table 1:** Demographic characteristics and vegetable intakes of the participants of the cross-sectional study.

Variables		Total		Male		Female	
	*n*	373		45		328	
		Mean±SD	range	mean±SD	range	mean±SD	range
Age	(y)	37.9±23.3	18–87	48.2±23.5	21–85	36.5±23.0	18–87
Height	(cm)	158.2±7.3	120–185	169.5±6.4	156–185	156.7±5.9	120–171
Weight	(kg)	52.9±8.7	34–87	66.2±7.6	50–87	51.0±7.1	34–80
Parity		-	-	-	-	0.6±1.0	0–4
Food consumption	(g/d)						
	cereal	245±155	0–720	253±144	0–540	244±157	0–720
	potato	25±54	0–600	39±51	0–150	23±54	0–600
	vegetable	245±207	0–1338	297±290	0–1338	239±196	0–1265
	fruits	57±105	0–545	113±167	0–545	50±94	0–520
	tea	143±250	0–1500	231±289	0–1000	133±244	0–1500
Insecticide use	(*n* of items)	0.3±0.8	0–5	0.2±0.7	0–3	0.3±0.8	0–5
		%		%		%	
Vegetable eating habit^a^	often	74.7%		73.9%		74.8%	
	sometimes	25.3%		26.1%		25.2%	
Drinking	current drinker	34.6%		75.6%		28.9%	
	ex-drinker	4.1%		11.1%		3.1%	
	non-drinker	61.4%		13.3%		68.0%	
Smoking	current smoker	0.8%		0.0%		0.9%	
	ex-smoker	7.3%		40.0%		2.8%	
	non-smoker	91.9%		60.0%		96.3%	

SD, standard deviation.

^a^ ‘Often’ means eating vegetables at least once a day.

### Reagents

Acetamiprid, imidacloprid, nitenpyram, thiacloprid, and thiamethoxam were obtained from AccuStandard (New Haven, CT, USA). Clothianidin, dinotefuran, and thiacloprid amide were obtained from Wako Pure Chemical Industries (Osaka, Japan). Desmethyl-acetamiprid and desmethyl-thiamethoxam were obtained from Sigma-Aldrich (St. Louis, MO, USA). Acetamiprid-d6 was obtained from Hayashi Pure Chemicals (Osaka, Japan). Acetamiprid-d3, clothianidin-d3, imidacloprid-d4, and thiamethoxam-d4 were obtained from Dr. Ehrenstorfer (Augsburg, Germany). Dinotefuran-d3 and thiacloprid-d4 were obtained from @rtMolecule (Poitiers, France) and C/D/N Isotopes (Pointe-Claire, Canada), respectively.

### Extraction of neonicotinoids from urine samples

Surrogate recovery standards (0.2 ng each of acetamiprid-d3, clothianidin-d3, imidacloprid-d4, thiacloprid-d4, and thiamethoxam-d4, and 2 ng of dinotefuran-d3) in acetonitrile were added to a 1 mL aliquot of a urine sample, then the sample was loaded onto a diatomite column (InertSep K-solute 2 mL; GL Sciences, Tokyo, Japan). The surrogate standards were not added to the urine samples collected in the deuterium-labeled neonicotinoid dosing study. The diatomite column was left for 10 min after the sample loading step was complete, and then the target analytes were eluted with 25 mL of dichloromethane over a period of 2 min. The eluate was rotary evaporated to approximately 10 mL then evaporated to approximately 1 mL under a stream of nitrogen. The extract was then passed through a Supelclean ENVI-Carb-II/PSA solid phase cartridge (containing 500 mg of each sorbent; Sigma-Aldrich). The target analytes were eluted using 10 mL of 20% (v/v) dichloromethane in acetonitrile over a period of 10 min. The extract was then evaporated to dryness under a stream of nitrogen and reconstituted in 30% methanol in water.

### Analysis and quality assurance

The extracts were analyzed using a Nexera liquid chromatography system (Shimadzu, Kyoto, Japan) coupled to a Triple Quad 6500 mass spectrometer (AB SCIEX, Framingham, MA, USA) with an atmospheric pressure electrospray interface, which was operated in positive ion mode. Separation was achieved using an Atlantis T3 column (100 mm long, 2.1 mm i.d., 3 μm particle diameter; Waters, Milford, MA, USA), which was kept at 40°C. The injection volume was 10 μL and the mobile phase flow rate was 200 μL/min. A gradient program with two mobile phases (acetonitrile and water containing 0.1% formic acid and 10 mM ammonium acetate) was used (S2 Table). A multiple reaction monitoring program with optimized parameters for each analyte was used to measure two product ions for each analyte (S2 Table).

Stock solutions of the standards were diluted and used to prepare a calibration curve with at least seven points for each analyte (S3 Table). The detection limit was defined as the mass of analyte producing a peak with a signal-to-noise ratio of 3 (S3 Table).

Acetamiprid, clothianidin, dinotefuran, imidacloprid, thiacloprid, and thiamethoxam were quantified using the internal standard method, using the deuterium-labeled analog of each analyte as the internal standard for that analyte. The external standard method was used for the other target analytes. The external standard method was used to analyze acetamiprid-d6, clothianidin-d3, dinotefuran-d3, and imidacloprid-d4 in the urine samples from the deuterium-labeled neonicotinoid dosing study. Desmethyl-acetamiprid-d3 was not commercially available, so it was quantified using the unlabeled desmethyl-acetamiprid calibration curve. The recoveries were evaluated by analyzing eight replicate fortified urine samples that originally contained low concentrations of the analytes. The recoveries of all the analytes were 64–100% (S3 Table). Procedural blanks were analyzed with each 16 samples (n = 29), and no neonicotinoid analytes were found in these blanks.

### Statistical analysis

All statistical analyses were performed using JMP software (Version 11; SAS Institute, Cary, NC, USA). Statistical significance was considered to be indicated when p<0.05. Each concentration below the detection limit was given a value of zero in the statistical analyses. The neonicotinoid concentrations in each urine sample were corrected for the creatinine concentration in the sample. The amount of creatinine excreted in the urine over 24 h was assumed to be 1.5 g for males and 1 g for females when calculating the daily amount of each neonicotinoid excreted from the spot urine sample data.[16] The mean amounts of the neonicotinoids that were excreted and the categorical lifestyle variables of the participants were investigated using the analysis of variance method (ANOVA) for variables with three categories and Student’s *t*-test for variables with two categories. Correlations between the amounts of the neonicotinoids that were excreted and the continuous characteristics of the participants were identified using the Pearson’s product moment correlation coefficient. Correlations between the neonicotinoid concentrations were also identified using the Pearson’s product moment correlation coefficient.

### Pharmacokinetic modeling

We developed a one-compartment pharmacokinetic model for clothianidin, dinotefuran, and imidacloprid and a two-compartment pharmacokinetic model for acetamiprid to describe the metabolic fates of these pesticides in urine (S1 Fig). We conducted an experiment in which deuterium-labeled neonicotinoids were orally ingested and their fates in the urine traced to allow these pharmacokinetic models to be developed. In this experiment, we assumed that the labeled neonicotinoid dose could be treated as a bolus, entering the body instantly after being administered (S2 Fig). We collected a urine sample from each participant every 24 h after dosing in this experiment.

We identified the statistical characteristics of acetamiprid, clothianidin, dinotefuran, and imidacloprid assuming that the pharmacokinetic behaviors of these compounds followed pharmacokinetic models that have previously been described.[17] We further assumed that the daily intakes of these compounds could be approximated as repeated daily bolus doses. The kinetic modeling is described in detail in S1 Method.

## Results

### Toxicokinetic parameters and estimated intakes of neonicotinoids

**Fig 2:**
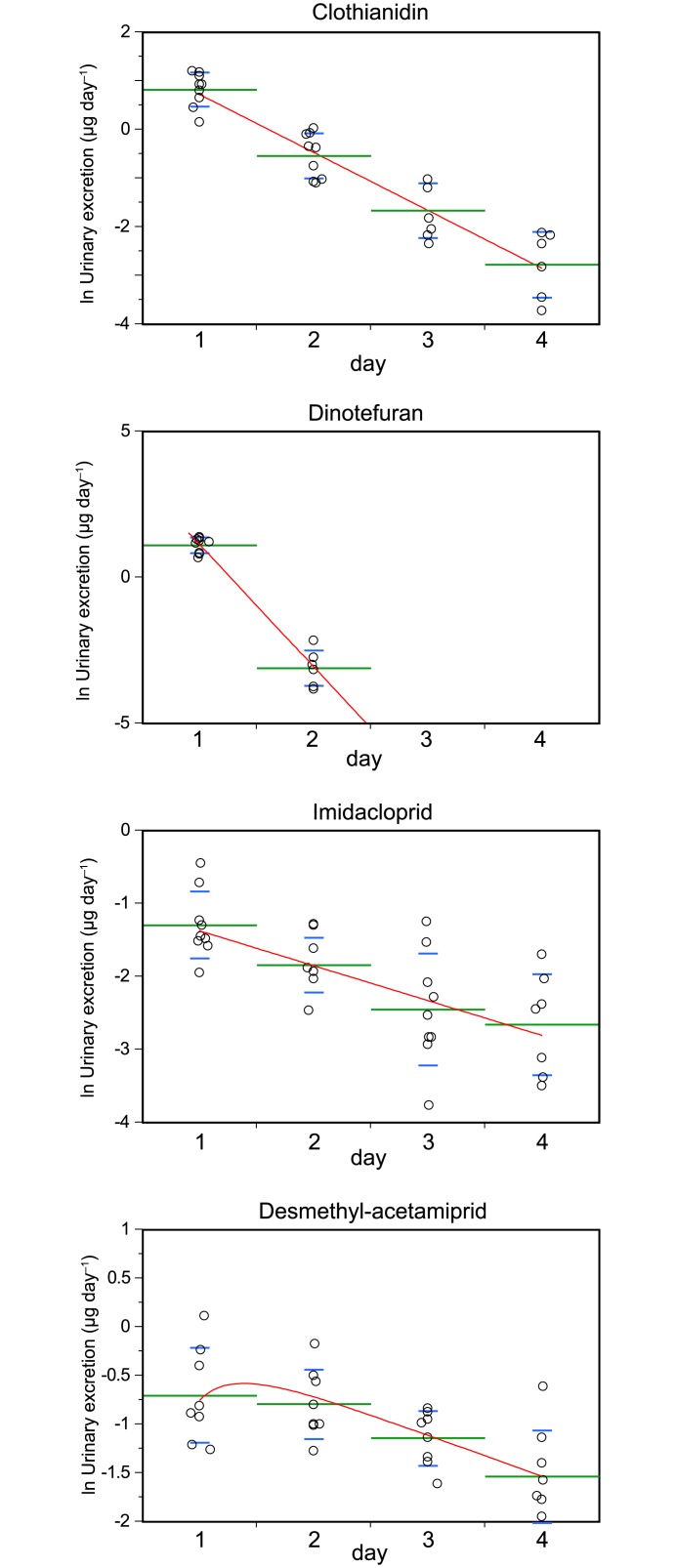
Amounts of the labeled compounds found to be excreted in the urine (μg d^−1^) in a 24 h period after a single dose was ingested (circles) and the model curves (red lines). The green bars are the means and the lower and upper blue whiskers are the standard deviations.

The labeled neonicotinoid concentrations in the urine increased when the participants ingested 5 μg of each deuterium-labeled neonicotinoid (Fig 2). Within 96 h of ingestion, 63.7% of the clothianidin had been recovered unchanged, and most of the dinotefuran was recovered within 1 day (92.8%; S4 Table). However, 12.7% of the ingested imidacloprid was excreted unchanged, and most of the acetamiprid that was ingested was metabolized to desmethyl-acetamiprid, which was eliminated more slowly than the other compounds that were used. Therefore, desmethyl-acetamiprid was used in the excretion model.

**Fig 3:**
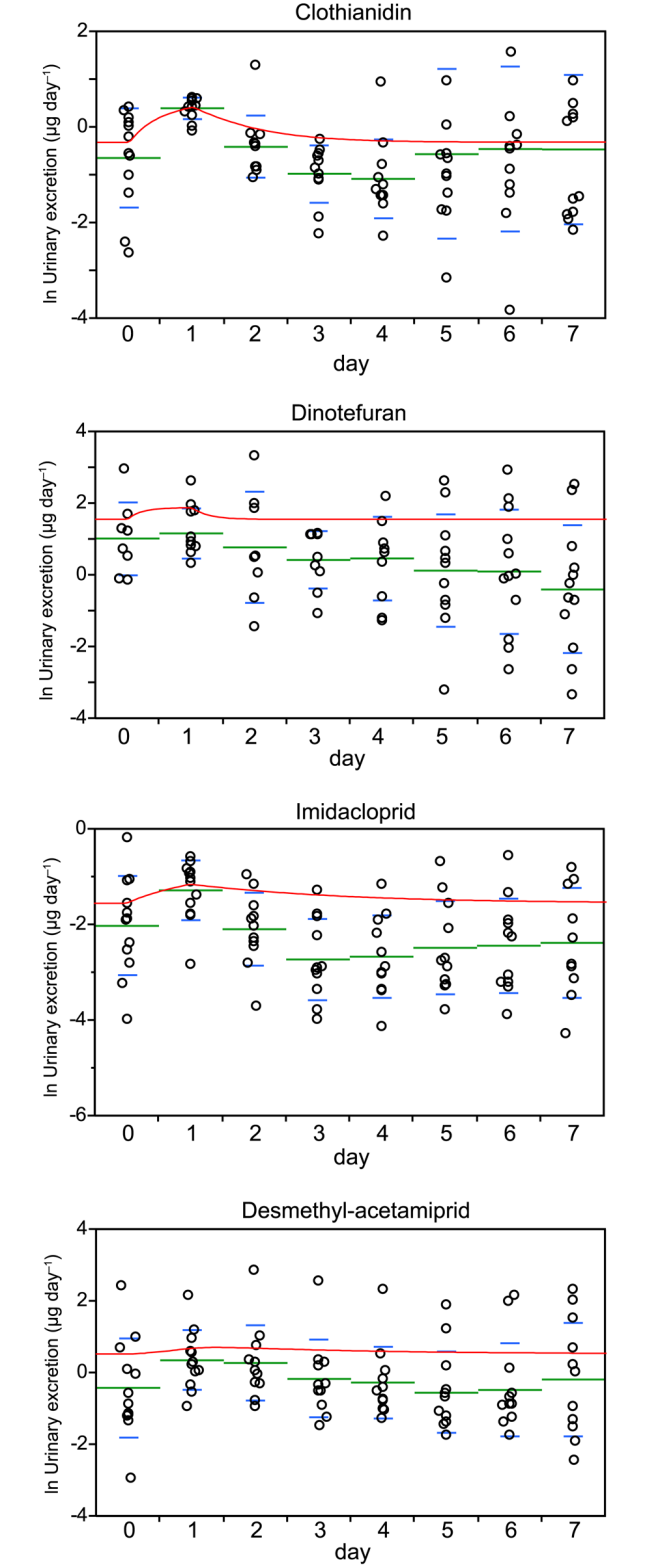
Amounts of the neonicotinoids excreted in urine during a 24 h period before (day 0) and after a single 2 μg dose was ingested (circles) and the model curves (red lines). The green bars are the means and the lower and upper blue whiskers are the standard deviations.

The urinary excretion time-courses of the test compounds over 96 h after the microdoses had been ingested were fitted to the following equations (details are given in S1 Method) to give the parameters r, α, and β (Table 2 and Fig 3).

**Table 2:** Parameters found for the pharmacokinetic models.

	*r*	*α* (d^−1^)	*T*_*1/2*_*α* (d)	*β* (d^−1^)	*T*_*1/2*_*β* (d)	*n*	*R*^2^
Clothianidin	0.596	1.2	0.58	—	—	31	0.88
Imidacloprid	0.133	0.479	1.45	—	—	34	0.46
Dinotefuran	0.899	4.2	0.17	—	—	15	0.96
Desmethyl-acetamiprid	0.586	3.08	0.23	0.419	1.65	32	0.42

The parameters for Eqs. 3 and 10 (S1 Method) were determined from the observed amounts of the deuterium-labeled chemicals excreted in the urine after a single ingested dose. *r* is the portion distributed into the compartment of interest (the area under the curve from zero to infinity). *α* and *β* are elimination rates and *T*_*1/2*_ is the half-life. The *R*^2^ values are the correlation coefficients for the relationships between the observed and modeled amounts excreted in the urine.

$U(N)=M\times r\times \left\{e^{-\alpha \times 24(N-1)}-e^{-\alpha \times 24N}\right\}$ for clothianidin, dinotefuran, and imidacloprid.

$U(N)=\frac{M\times r}{\frac{1}{\alpha }-\frac{1}{\beta }}\text{​​​​​​​​}\times [\frac{1}{\alpha }\left\{e^{-\alpha \times 24(N-1)}-e^{-\alpha \times 24\;N}\right\}-\frac{1}{\beta }\left\{e^{-\beta \times 24(N-1)}-e^{-\beta \times 24\;N}\right\}]$ for desmethyl-acetamiprid.

In these equations, *U(N)* is the amount of the neonicotinoid of interest excreted in urine between N−1 days and N days, *r* is the proportion excreted in urine in the study (the area under the curve from time zero to an infinite number of days), *α* and *β* are elimination rates, and *T*_*1/2*_ is the half-life. The kinetic parameter *r* therefore indicates the fraction of the ingested compound that could be biologically monitored, and the remainder of the ingested compound would have been converted into unidentified metabolites. Imidacloprid had an *r* of 0.133, indicating that a large proportion of the ingested imidacloprid would have been converted into unidentified metabolites. Halves of clothianidin and acetamiprid were also metabolized into unidentified metabolites.

The urinary excretion time courses for the 2 μg unlabeled neonicotinoid bolus tests showed that the mean concentrations of clothianidin, imidacloprid, and desmethyl-acetamiprid had increased after one day. The dinotefuran concentrations increased only slightly because the baseline concentrations were high. The time courses were compared with the toxicokinetic models that had been developed, and the models were found to fit the data well (the R^2^ values indicated that the fits were significant), as shown in Fig 3 and S5 Table. The neonicotinoid concentrations in each participant were assumed to be at steady state before the bolus was administered, and the daily acetamiprid, clothianidin, dinotefuran, and imidacloprid intakes were estimated to be 2.93 ± 12.4, 1.26 ± 1.12, 5.18 ± 6.40, and 1.58 ± 3.37 μg, respectively, using Eqs 13, 15, and 18 (S1 Method).

### Urinary excretion and estimated intakes of neonicotinoids in healthy individuals

**Fig 4:**
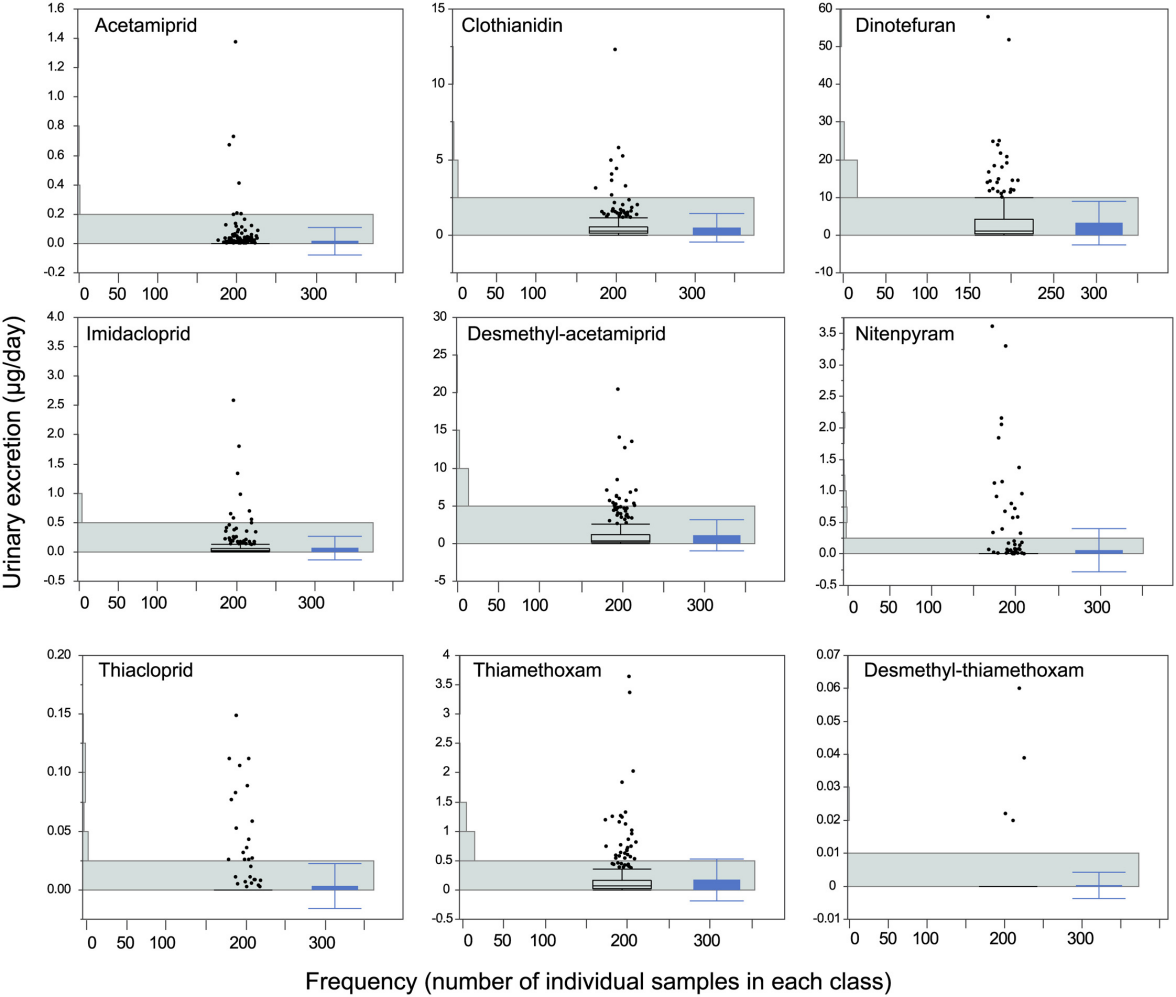
Distribution of neonicotinoid excretion rates in healthy adults (μg/d). The horizontal bars show the frequencies (the *x*-axis is the number of individual samples in each class). The black line boxes at the center of each graph show the first, second, and third quartiles. The lower whisker indicates the lowest value within the −1.5 interquartile range of the first quartile. The upper whisker indicates the highest value within the +1.5 interquartile range of the third quartile. Outlying values are shown as dots. The blue bars on the right-hand sides of the graphs are the means and standard deviations.

Clothianidin, desmethyl-acetamiprid, dinotefuran, imidacloprid, and thiamethoxam were detected in more than half of the samples that were analyzed (Table 3). The average amounts excreted were 3.29 μg/d for dinotefuran, 1.14 μg/d for desmethyl-acetamiprid, 0.51 μg/d for clothianidin, and 0.07 μg/d for imidacloprid. Toxicokinetic models were not developed for nitenpyram, thiacloprid, and thiamethoxam, but these compounds were found in the urine samples. Thiamethoxam was frequently detected, and an average of 0.18 μg/d was excreted. However, desmethyl-thiamethoxam, a metabolite of thiamethoxam, was detected in only four samples. Thiacloprid amide was not detected in any of the samples. The amounts of each neonicotinoid excreted were not normally distributed but had skewed distributions (Fig 4).

**Table 3:** Amounts of neonicotinoid pesticides excreted in the urine (*n* = 373) and the estimated daily intakes.

	Excretion in urine (μg/d)^a^						
	*n*>LOD (%)	mean±SD	median	75%ile	90%ile	max	
Acetamiprid	91 (24.4%)	0.02±0.09	n.d.	n.d.	0.04	1.38	
Clothianidin	360 (96.5%)	0.51±0.95	0.27	0.53	1.15	12.3	
Dinotefuran	348 (93.3%)	3.29±5.80	1.02	4.20	8.63	57.9	
Imidacloprid	286 (76.7%)	0.07±0.20	0.03	0.06	0.14	2.59	
Nitenpyram	44 (11.8%)	0.07±0.34	n.d.	n.d.	0.01	3.62	
Thiacloprid	29 (7.8%)	0.004±0.019	n.d.	n.d.	n.d.	0.22	
Thiamethoxam	343 (92.0%)	0.18±0.36	0.07	0.16	0.41	3.64	
Desmethyl-acetamiprid	373 (100%)	1.14±2.07	0.40	1.16	2.92	20.48	
Desmethyl-thiamethoxam	4 (1.1%)	0.0004±0.0040	n.d.	n.d.	n.d.	0.06	
Thiacloprid amide	0 (0%)	-	-	-	-	-	
	Intake (μg/d)^b^						ADI
	mean±nominal SD	theoretical SD^c^	median	75%ile	90%ile	max	mg/(kg BW d)
Acetamiprid^d^	1.94±3.53	6.03	0.67	1.97	4.98	34.9	0.071
Clothianidin	0.86±1.59	2.67	0.46	0.89	1.93	20.7	0.097
Dinotefuran	3.66±6.45	7.18	1.13	4.67	9.60	64.5	0.22
Imidacloprid	0.53±1.52	11.41	0.19	0.43	1.06	19.5	0.057

LOD, limit of detection;

SD, standard deviation; n.d., not detected;

ADI, acceptable daily intake

^a^ Excretion in the urine was calculated assuming that the amount of creatinine excreted each day was 1.5 g for males and 1 g for females.

^b^ The intake was calculated from the portion distributed into the ‘r’ compartment, derived from the dosing study.

^c^ The theoretical SD was calculated using Eqs 15 and 18 (S1 Method).

^d^ The acetamiprid intake was estimated from the amount of desmethyl-acetamiprid excreted.

The amounts excreted were converted into intakes using the parameter r from the toxicokinetic models, and the estimated daily intakes were found to be 3.66 μg/d for dinotefuran, 1.94 μg/d for acetamiprid, 0.86 μg/d for clothianidin, and 0.53 μg/d for imidacloprid. Theoretical standard deviations for the estimated intakes were calculated using Eqs 15 and 18 (S1 Method). The theoretical standard deviation for the estimated imidacloprid intake was rather high because imidacloprid was found to be poorly excreted in urine. The neonicotinoid with the highest estimated intake was dinotefuran, at 64.5 μg/d, which is about 10% of the intake estimated in previous risk assessments and less than 1% of the acceptable daily intake in Japan.[8]

### Factors related to neonicotinoid concentrations

Five of the neonicotinoids were frequently detected, and the associations between their concentrations and diet and lifestyle variables were examined (Table 4). The clothianidin, desmethyl-acetamiprid, dinotefuran, and thiamethoxam concentrations correlated slightly with age and parity (Table 4). The clothianidin, desmethyl-acetamiprid, dinotefuran, and imidacloprid concentrations correlated with fruit intake the day before the samples were collected. The dinotefuran and imidacloprid concentrations weakly correlated with vegetable intake the day before the samples were collected, and the dinotefuran concentration also correlated with the cereal intake. The neonicotinoid concentrations did not significantly correlate with the sex of the participants, their tea intake, or the amount of pesticide they used. The participants who ate vegetables “often” tended to have higher neonicotinoid concentrations in their urine than did the other participants (those who ate vegetables ‘sometimes’), but the differences were not statistically significant (p>0.05, Student’s *t*-test). The clothianidin, dinotefuran, and imidacloprid concentrations were associated with the drinking or smoking habits of the participants (p<0.05, ANOVA).

**Table 4:** Correlations between amounts neonicotinoids excreted and the characteristics of the participants.

	Clothianidin		Desmethyl-acetamiprid		Dinotefuran		Imidacloprid		Thiamethoxam	
	r	p value	r	p value	r	p value	r	p value	r	p value
Age	0.21	**<.0001**	0.19	**0.0002**	0.21	**<.0001**	0.23	**<.0001**	0.06	0.23
Height	0.01	0.79	−0.01	0.81	0.05	0.29	−0.10	0.06	0.01	0.90
Weight	0.01	0.88	0.01	0.81	0.08	0.15	−0.10	0.07	−0.05	0.40
Parity	0.25	**<.0001**	0.24	**<.0001**	0.24	**<.0001**	0.17	**0.004**	0.07	0.24
Food consumption										
cereal	0.06	0.28	−0.05	0.41	0.18	**0.002**	−0.02	0.74	0.02	0.71
potato	0.03	0.58	0.004	0.94	0.04	0.47	−0.007	0.91	−0.05	0.36
vegetable	0.09	0.14	0.06	0.28	0.12	**0.03**	0.27	**<.0001**	0.03	0.56
fruit	0.14	**0.02**	0.14	**0.02**	0.14	**0.02**	0.17	**0.005**	0.004	0.94
tea	0.10	0.10	0.05	0.40	0.10	0.13	0.11	0.09	0.04	0.55
Insecticide use	−0.05	0.38	−0.05	0.37	−0.07	0.24	0.01	0.81	−0.004	0.94
	mean±SD	p value^a^	mean±SD	p value	mean±SD	p value	mean±SD	p value	mean±SD	p value
Sex										
male	0.7±1.8	0.16	1.3±3.1	0.48	4.1±9.0	0.34	0.07±0.20	0.98	0.17±0.23	0.86
female	0.5±0.8		1.1±1.9		3.2±5.2		0.07±0.20		0.18±0.37	
Vegetable eating habits^b^										
often	0.6±1.1	0.08	1.2±2.2	0.055	3.2±4.4	0.12	0.06±0.10	0.07	0.19±0.43	0.07
sometimes	0.3±0.3		0.6±1.0		2.3±3.1		0.03±0.06		0.09±0.11	
Drinking										
current drinker	0.5±0.6	**0.02**	1.2±1.8	0.20	3.9±8.1	0.23	0.11±0.31	**0.02**	0.21±0.42	0.29
ex-drinker	1.2±3.1		2.0±5.2		4.0±4.6		0.03±0.03		0.21±0.23	
non-drinker	0.5±0.8		1.0±1.8		2.9±4.1		0.05±0.11		0.15±0.33	
Smoking										
current smoker	0.13±0.15	0.77	0.31±0.31	0.70	0.07±0.12	**0.005**	0.05±0.04	0.66	0.04±0.04	0.72
ex-smoker	0.5±0.5		1.3±1.2		6.7±11.6		0.11±0.25		0.15±0.19	
non-smoker	0.5±1.0		1.1±2.1		3.1±5.0		0.07±0.20		0.18±0.37	

r, Pearson’s product moment correlation coefficient for continuous characteristics; SD, standard deviation.

^a^ Analysis of variance or Student’s *t*-test for categorical characteristics.

^b^ ‘Often’ means eating vegetables at least once a day.

### Correlations between neonicotinoid pesticides concentrations

The correlation coefficient for the relationship between the clothianidin and desmethyl-acetamiprid concentrations was 0.54 (the highest for the relationships between the pesticide concentrations), and a correlation coefficient of 0.49 was found both for the relationship between the dinotefuran and nitenpyram concentrations and for the relationship between the thiacloprid and nitenpyram concentrations (Table 5). The acetamiprid concentration weakly correlated with the desmethyl-acetamiprid and imidacloprid concentrations. The clothianidin concentration weakly correlated with the imidacloprid, thiacloprid, and thiamethoxam concentrations.

**Table 5:** Parametric correlation coefficients for the relationships between the neonicotinoid concentrations.

	Acetamiprid	Clothianidin	Dinotefuran	Imidacloprid	Nitenpyram	Thiacloprid	Thiame thoxam	Desmethyl- acetamiprid	Desmethyl- thiamethoxam
Clothianidin	**0.15**	1							
Dinotefuran	0.09	**0.19**	1						
Imidacloprid	**0.31**	**0.26**	**0.12**	1					
Nitenpyram	**0.11**	**0.13**	**0.49**	**0.30**	1				
Thiacloprid	0.07	**0.21**	**0.26**	**0.30**	**0.49**	1			
Thiamethoxam	0.04	**0.33**	**0.15**	0.09	0.07	0.05	1		
Desmethyl- acetamiprid	**0.30**	**0.54**	**0.14**	**0.26**	0.05	0.07	**0.18**	1	
Desmethyl- thiamethoxam	−0.02	−0.02	0.02	−0.03	−0.02	−0.02	−0.03	−0.04	1

Bold figures indicate statistically significant results, determined using Pearson’s product moment correlation coefficient (p<0.05).

## Discussion

Clothianidin and dinotefuran were mostly recovered unmetabolized in the urine collected in the toxicokinetics study (63.7% and 92.8% within 96 h, respectively). The proportion excreted in urine was smaller for acetamiprid and imidacloprid (2.6% and 12.7% within 96 h, respectively) than for clothianidin and dinotefuran. Acetamiprid was preferentially metabolized to desmethyl-acetamiprid, which was excreted in the urine (30.7% within 96 h). The acetamiprid and imidacloprid concentrations in the urine were therefore low even though the estimated dietary intakes of these compounds were comparable to the estimated dietary intake of clothianidin. The excretion kinetics were modeled, and the *R*^*2*^ values were 0.42–0.96, justifying the use of clothianidin, dinotefuran, imidacloprid, and desmethyl-acetamiprid as biological monitoring markers. However, the kinetic parameter *r* ranged from 0.133 to 0.899, indicating that there were substantial amounts of unidentified metabolites. We did not include these unidentified metabolites in the toxicokinetic and risk assessment evaluations. We assumed that exposure to neonicotinoids would be represented by the presence of the unmetabolized compounds and that the toxicological profiles would be similar to those found in animal experiments. Another risk assessment would be required if metabolites with long half-lives and high toxicities to humans were present in the applied products. Indeed, two imidacloprid metabolites, 5-hydroxyimidacloprid and olefin, are as toxic as the parent compound to honeybees, *Apis mellifera*.[18,19] Delayed and time-cumulative imidacloprid toxicity in honeybees and other insects could be caused by the metabolites being only slowly excreted.[20] Exposure to metabolites and the toxicities of the metabolites to humans were not within the scope of this study, but should be investigated in future studies.

Clothianidin, desmethyl-acetamiprid, dinotefuran, imidacloprid, and thiamethoxam were frequently detected in the cross-sectional survey samples. Nitenpyram and thiacloprid were also found in some samples, but it is unclear whether this was because dietary intakes of these chemicals by the participants were low or because they are easily metabolized. In a toxicokinetics study using mice, 46% of the nitenpyram dose was recovered in urine within 24 h but only 1.3% of the thiacloprid dose was recovered in urine, and 12% of the thiacloprid was found to be converted into the 6-chloropyridine carboxylic acid.[12] If the kinetics of neonicotinoids are similar in humans and mice, the intake of nitenpyram by Japanese adults is unlikely to be high, but there was a large degree of uncertainty in the estimated intake of thiacloprid. In mice, 27% of the thiamethoxam dose was found to be excreted in urine and 11% was found to be converted into clothianidin.[11] There was a weak correlation between clothianidin and thiamethoxam in our study (r = 0.33, Table 5), suggesting that part of the clothianidin could have been derived from thiamethoxam. Desmethyl-thiamethoxam was detected in only 1% of the samples analyzed, so the demethylation of thiamethoxam was considered to be a minor metabolic pathway, as has been found *in vitro* previously.[21] Thiacloprid amide is a persistent metabolite of thiacloprid in soil,[22] but none of our urine samples contained detectable thiacloprid amide concentrations. Thiacloprid amide could, therefore, be further metabolized in humans.

Age and parity were associated with the neonicotinoid concentrations in the urine samples, but it is possible that these associations could have been confounded by the vegetable intake. Indeed, vegetable intake positively correlated with age and parity in the study population (r = 0.38, p<0.0001 for age and r = 0.41, p<0.0001 for parity). Fruit intake was associated with the concentrations of various neonicotinoids, but cereal intake correlated only with the dinotefuran concentration. This heterogeneity in the correlations found may have been caused by differences in the amounts of the neonicotinoids that are applied to different crops. Dinotefuran is widely used in household insecticides, but no correlation was found between household insecticide use and the dinotefuran concentration. Information on the ingredients of the insecticides the participants used were not collected in the questionnaire, so the number of insecticides used did not necessarily reflect the use of neonicotinoids. Nevertheless, neonicotinoids are not volatile and are unlikely to be inhaled in the home environment. As described above, exposure through food consumption affected the concentrations in urine. Correlations were found between the concentrations of some of the neonicotinoids even though they did not have precursor—metabolite relationships. It is possible that these pesticides have been used as mixtures or that residues of these pesticides in soil were incorporated in crops.

The study presented here had several limitations. The toxicokinetics of neonicotinoid pesticides were investigated after each participant ingested a single dose of the test compounds, and the results were extrapolated to allow the daily intake to be estimated. Repeated doses might affect the toxicokinetics of the chemicals, while the participants of the toxicokinetic studies were exposed to neonicotinoids at comparable concentrations before they ingested the doses for the experiments. More females than males enrolled in the cross-sectional survey. Even though no differences in neonicotinoid concentrations between the sexes were found, it is possible that the estimates were biased because of the unrepresentative sex ratio of the participants. Another potential source of bias is that the urine samples were all collected in urbanized cities in Kyoto Prefecture. Vegetables and other products are distributed to major markets throughout Japan, but there could be differences in the amounts and types of pesticides applied to produce in different areas. However, the 373 participants were recruited from young and old populations, which may have decreased the potential for study bias. Whatever the case may be, fruits and vegetables were found to be the main sources of human exposure to neonicotinoids.

In conclusion, a human pharmacokinetic model was established for four neonicotinoids. Most of the ingested dinotefuran was excreted in urine, whereas most of the acetamiprid and imidacloprid was metabolized, as was half of the clothianidin. The major metabolite of acetamiprid, desmethyl-acetamiprid, was slowly excreted. These compounds, in urine, could be used as reliable biological monitoring markers of exposure to the compounds, and the daily intakes of these neonicotinoids could be estimated from their concentrations in urine. The exposure of the general population of Japan to these four neonicotinoids was evaluated. Most people in Japan are frequently exposed to neonicotinoid residues in the fruit and vegetables they consume, but the daily intakes of neonicotinoids are well below current Japanese acceptable daily intakes. Exposure to unidentified metabolites should be studied in future work, and risk assessments of these metabolites should be performed.

## Supporting Information

S1 Fig Compartmental models for the toxicokinetics of the neonicotinoids. (DOCX)

S2 Fig Illustration of the assumption used in the kinetic models that each deuterium-labeled neonicotinoid was administered as a single bolus and entered the body instantaneously after ingestion, and an illustration of the urinary excretion of the neonicotinoid in urine after such a dose. (DOCX)

S1 Method (DOCX)

S1 Table Demographic characteristics of the participants of the microdose study. (DOCX)

S2 Table LC-MS/MS conditions used to determine the neonicotinoids. (DOCX)

S3 Table Calibration curves, detection limits, and recoveries for the neonicotinoids. (DOCX)

S4 Table Amounts of neonicotinoids excreted in urine over 96 h after a 5 μg microdose of each deuterium-labeled compound was ingested, and the percentage of the initial parent compound dose that was recovered. (DOCX)

S5 Table Estimated daily intakes of neonicotinoids found in the microdose. (DOCX)
