# Supplementary material for: Biological Monitoring of Human Exposure to Neonicotinoids Using Urine Samples, and Neonicotinoid Excretion Kinetics
Source: PLoS One. 2016 Jan 5;11(1):e0146335. doi: 10.1371/journal.pone.0146335 (PMC4701477; doi:10.1371/journal.pone.0146335)
Supplement: S1 Fig — (DOCX) [file pone.0146335.s001.docx]

**S1 Fig**. Compartmental models for the toxicokinetics of the neonicotinoids
