# Supplementary material for: Biological Monitoring of Human Exposure to Neonicotinoids Using Urine Samples, and Neonicotinoid Excretion Kinetics
Source: PLoS One. 2016 Jan 5;11(1):e0146335. doi: 10.1371/journal.pone.0146335 (PMC4701477; doi:10.1371/journal.pone.0146335)
Supplement: S2 Fig — (DOCX) [file pone.0146335.s002.docx]

**S2 Fig**. Illustration of the assumption used in the kinetic models that each deuterium-labeled neonicotinoid was administered as a single bolus and entered the body instantaneously after ingestion, and an illustration of the urinary excretion of the neonicotinoid in urine after such a dose
