# Supplementary material for: Biological Monitoring of Human Exposure to Neonicotinoids Using Urine Samples, and Neonicotinoid Excretion Kinetics
Source: PLoS One. 2016 Jan 5;11(1):e0146335. doi: 10.1371/journal.pone.0146335 (PMC4701477; doi:10.1371/journal.pone.0146335)
Supplement: S1 Method — (DOCX) [file pone.0146335.s003.docx]

**S1 Method**

1. **Pharmacokinetic models for deuterium-labeled acetamiprid, clothianidin, dinotefuran, and imidacloprid**

1. *One compartment pharmacokinetic model*

Most of the clothianidin, dinotefuran, and imidacloprid in the doses were found to be excreted in the urine as the parent compounds, so we used a one-compartment model for these compounds. The amounts excreted in the urine over 24 h were calculated as follows:

$V\frac{\mathrm{dC}}{\mathrm{dt}}=M\times r-k\times c$, (1)

where V is the volume distribution, c is the concentration, M is the amount administered as a single dose, r is the portion distributed into the compartment of interest, and k is the clearance rate (see Supporting Information, Figure S1). Note that the dose was administered as a bolus, so $M\times r$ will disappear from Eq. 1, giving Eq. 2.

$V\frac{\mathrm{dC}}{\mathrm{dt}}=-k\times c$ (2)

The initial condition is given by $c1(0)=\frac{M\times r}{V}$. We can then obtain,

$U\left( N \right)=\int_{24(N-1)}^{24N} k\times c dt$, (3)

and

, (4)

where α is equal to $\frac{k}{V}$and U(N) is the amount of a neonicotinoid excreted in urine between N−1 days and N days, where $N\geq1.$

1. *Two compartment pharmacokinetic model*

In the two compartment model, the first compartment represents the kinetics of the parent compound and the second the kinetics of the metabolite. This model was used to represents the kinetics of acetamiprid and its metabolite desmethyl-acetamiprid.

Two differential equations (Supporting Information, Figure S2), shown in Eqs. 5–7, were used.

$V1\frac{dc1}{\mathrm{dt}}=M\times r-k1\times c1-k2xc1$ (5)

Note that $M\times r$ will disappear soon after dosing, giving Eq. 6.

$V1\frac{dc1}{\mathrm{dt}}=-k1\times c1-k2xc1$ (6)

$V2\frac{dc2}{\mathrm{dt}}=-k2\times C1-k3\times c2$ (7)

We can assume that c1(0)=$\frac{M\times r}{V1}$ and c2(0)=0 at the initial conditions. Excretion in the urine is given as shown in Eq. 8 because

$U1\left( N \right)=\int_{24(N-1)}^{24N} k1\times c1 dt$.

$U1\left( N \right)=M\times r\times\frac{k1}{k1+k2}\times({e^{-\frac{k1+k2}{V1}\times24(N-1)}-e^{-\frac{k1+k2}{V1}\times24N})}$ (8)

Likewise we have the amount of metabolite excreted in the urine,

$U2\left( N \right)=\left( \frac{M\times r\times k2}{k3\times V1-\left( k1+k2 \right)\times V2} \right)\times\left[ \frac{k3\times V1}{k1+k2}\times\left( e^{-\frac{\left( k1+k2 \right)\times24\left( N-1 \right)}{V1}}-e^{-\frac{\left( k1+k2 \right)\times24N}{V1}} \right)- V2\times\left( e^{\frac{-k3\times24\left( N-1 \right)}{V2}}-e^{\frac{-k3\times24N}{V2}} \right) \right]$ ,

where N≥1.

We can simplify the above equation to give Eq. 9,

$=\frac{\left( k2\times k3\times M\times r \right)}{k3\times\left( k1+k2 \right)}\times\frac{1}{\left( \frac{1}{\alpha}-\frac{1}{\beta} \right)}\times\left[ \frac{1}{\alpha} \left( {e^{-\alpha\times24}}^{(N-1)}-{e^{-\alpha}}^{\times24N} \right)- \frac{1}{\beta}\left( {e^{-\beta}}^{\times24(N-1)}-{e^{-}}^{\beta\times24N} \right) \right]$ $-----Eq9$, (9)

where $\alpha=\frac{k1+k2}{V1} , \beta=\frac{k3}{V2} .$

We let the total amount excreted in urine on the Nth day be

$U(N)=U1(N)+U2 (N)$. (10)

When k1 << k2, U1 >> U2 and, therefore, U ≈ U2, allowing Eq. 11 to be derived.

$\mathrm{When}k1\ll k2, then U1\ll U2 and thus U\cong U2. Thus we will obatin Eq11.$

(11)

In fact, the bulk of the acetamiprid was excreted as desmethyl-acetamiprid (see the main manuscript).

1. **Statistical characteristics of the acetamiprid, clothianidin, dinotefuran, and imidacloprid data**

*1. Assumptions*

If we assume that the ith individual has a daily intake of M(i,j) and excretes U(i,j) in urine at the jth sampling point, we can assume that M(i,j) is made up of E[M(i,j)] and V[M(i,j)]. We can then assume that E[M(i,j)]= E(M) and V[M(i,j)]=V(M), irrespective of i and j.

These assumptions simply insist that the daily intake has a single mean and variance common to all subjects on any day. We can then introduce USt, the daily amount excreted in the urine when repeated doses occur (i.e., excretion in urine at steady state). With these assumptions we can obtain the relationships E[USt(i,j)]= E(USt) and V[USt(i,j)]=V(USt), where USt is the amount excreted in urine corresponding to an intake of M. In the following discussion we will consider how we can correlate E(M) and V(M) with E(USt) and V(USt).

*2 Estimating the mean and variance for the one compartment model*

To investigate the statistical characteristics we will define the stochastic process, [$M\infty,・・・・Mj,・・・Mi, ・・・Mo]$, where Mo is the intake on day 0, i.e., the sampling day, and Mj is the intake j days before the sampling point.

We can then derive the equation

$\mathrm{USt}=M0\times r\times\left( 1-a \right)+M1\times r\times\left( 1-a \right)a+・・・Mi\times r\times\left( 1-a \right)a^{i}+・・・$

$Mj\times r\times\left( 1-a \right)a^{j}+・・・・・$

$USt=\sum_{0}^{\infty} Mjr\left( 1-a \right)\times a^{j}$, (12)

where $a=e^{-aT}$, $Mi\times r\times\left( 1-a \right)a^{i}$, or $Mj\times r\times\left( 1-a \right)a^{j}$ represents carryover caused by dietary intake i or j days before the urine was collected. Note that Mi and Mj are independent random variables. If the daily intake is of interest, T is equal to 24 h. We can then obtain Eq. 13 from Eq. 12.

$E\left[ \mathrm{USt} \right]$= E$\left( Mr(1-a)\sum_{0}^{\infty} a^{i})=r \times E\left[ M \right] \right.$

(13)

We will next consider the variance of the daily intake. We can obtain the variance of the amount excreted in urine through our observations. In the following discussion we will define the relationship between the intake variance and the variance of the amount excreted in urine.

The variance of USt, $V\left[ USt \right],$is given by

$$Ｖ(USt)=(\sum_{0}^{\infty} r^{2}\left( 1-a \right)^{2}\times a^{2j})\times V(M)$$

$$=r^{2}\times\left( 1-a \right)^{2}\times\frac{1}{\left( 1-a^{2} \right)}\times V(M)$$

$=r^{2}\times\frac{1-a}{1+a}\times V\left( M \right)$, (14)

where $a=e^{-aT}.$ Note that Mj and Mi are independent. Therefore, we can obtain Eq. 15.

$V\left( M \right)=\frac{1+e^{-aT}}{r^{2}\left( 1-e^{-aT} \right)}V(USt)$ (15)

We can estimate the variance V(M) from Eq. 15 for the neonicotinoids for which we used the one-compartment pharmacokinetic model.

*3. Estimating the variance for the two compartment model*

Similar logic can be applied to E[USt] in the two-compartment model, giving

$E\left[ USt \right]=r\times E\left[ M \right]$.

To estimate the variance we will again consider the stochastic process. A minor portion of the acetamiprid is excreted in urine as the parent compound, so we can assume that k2 can be calculated as shown below.

$$USt=M0\times r\times\left( \frac{1}{L} \right)\times\left[ \frac{1}{a}\times\left( 1-a \right)-\frac{1}{\beta}\left( 1-b \right) \right]+・・・$$

$Mi\times r\times\left( \frac{1}{L} \right)\times\left[ \frac{1}{a}\times\left( 1-a \right)a^{i}-\frac{1}{\beta}\left( 1-b \right)b^{i} \right]+\ldots Mj\times r\times\left( \frac{1}{L} \right)\times\left[ \frac{1}{a}\times\left( 1-a \right)a^{j}-\frac{1}{\beta}\left( 1-b \right)b^{j} \right]+\ldots$ (16)

In Eq. 16 L= $\frac{1}{a}-\frac{1}{\beta}\mathrm{and}\alpha=\frac{k1+k2}{V1} , \beta=\frac{k3}{V2} , a= e^{-aT}and$b=$e^{-\beta T}.$

$V\left[ USt \right]=V\left\{ \sum_{i=0}^{\infty} \left( Mi\times r\times\left( \frac{1}{L} \right)\times\left[ \frac{1}{a}\times\left( 1-a \right)a^{i}-\frac{1}{\beta}\left( 1-b \right)b^{i} \right] \right) \right.$

$$=\left\{ \left. \sum_{i=0}^{\infty} \left( r\times\left( \frac{1}{L} \right)\times\left[ \frac{1}{a}\times\left( 1-a \right)a^{i}-\frac{1}{\beta}\left( 1-b \right)b^{i} \right] \right)^{2} \right\}V\left( M \right) \right.$$

$$=\left\{ \left. \frac{r^{2}}{L^{2}} \right\}\left. \left\{ \frac{1}{a^{2}} \right.\left( \frac{1-e^{-aT}}{1+e^{-aT}} \right)+\frac{1}{\beta^{2}}\left( \frac{1-e^{-\beta T}}{1+e^{-\beta T}} \right)-\frac{2}{a\beta}\frac{(1-e^{-aT})(1-e^{-\beta T})}{(1-e^{-aT-\beta T})} \right\}V\left( M \right) \right.$$

(17)

$$V(M)=\frac{V[USt]}{\left\{ \left. \frac{r^{2}}{L^{2}} \right\}\left. \left\{ \frac{1}{a^{2}} \right.\left( \frac{1-e^{-aT}}{1+e^{-aT}} \right)+\frac{1}{\beta^{2}}\left( \frac{1-e^{-\beta T}}{1+e^{-\beta T}} \right)-\frac{2}{a\beta}\frac{(1-e^{-aT})(1-e^{-\beta T})}{(1-e^{-aT-\beta T})} \right\} \right.}$$

$$]$$

(18)

We can estimate the variance V(M) from Eq. 18 for the neonicotinoids for which we used the two-compartment pharmacokinetic model.
