# Supplementary material for: Biological Monitoring of Human Exposure to Neonicotinoids Using Urine Samples, and Neonicotinoid Excretion Kinetics
Source: PLoS One. 2016 Jan 5;11(1):e0146335. doi: 10.1371/journal.pone.0146335 (PMC4701477; doi:10.1371/journal.pone.0146335)
Supplement: S1 Table — (DOCX) [file pone.0146335.s004.docx]

| **S1 Table.** Demographic characteristics of the participants of the microdose study | | | | | | | |
| --- | --- | --- | --- | --- | --- | --- | --- |
| Variables |  | Total |  | Male |  | Female |  |
|  | *n* | 12 |  | 6 |  | 6 |  |
|  |  | Mean±SD | Range | mean±SD | Range | mean±SD | Range |
| Age | (y) | 45.3±13.9 | 24–65 | 45.8±12.7 | 34–62 | 44.7±16.1 | 24–65 |
| Height | (cm) | 161.5±8.5 | 148–176 | 167.3±6.6 | 156–176 | 155.6±5.6 | 148–162 |
| Weight | (kg) | 60.4±14.3 | 42–86 | 70.7±12.3 | 55–86 | 50.1±6.9 | 34–80 |
| Parity |  | - | - | - | - | 0.8±1.3 | 0–3 |
| Drinking | current drinker | 58.3% |  | 66.7% |  | 50.0% |  |
|  | ex-drinker | 0% |  | 0% |  | 0% |  |
|  | non-drinker | 41.7% |  | 33.3% |  | 50.0% |  |
| Smoking | current smoker | 16.7% |  | 33.3% |  | 0% |  |
|  | ex-smoker | 25.0% |  | 50.0% |  | 0% |  |
|  | non-smoker | 58.3% |  | 16.7% |  | 100% |  |
| SD, standard deviation. | | | | | | | |
