# Supplementary material for: Biological Monitoring of Human Exposure to Neonicotinoids Using Urine Samples, and Neonicotinoid Excretion Kinetics
Source: PLoS One. 2016 Jan 5;11(1):e0146335. doi: 10.1371/journal.pone.0146335 (PMC4701477; doi:10.1371/journal.pone.0146335)
Supplement: S2 Table — (DOCX) [file pone.0146335.s005.docx]

| **S2 Table.** LC-MS/MS conditions used to determine the neonicotinoids | | | | |  |  |
| --- | --- | --- | --- | --- | --- | --- |
| Liquid chromatography | |  |  |  |  |  |
|  | Instrument | SHIMADZU Nexera | |  |  |  |
|  | Column | Atlantis T3 (2.1 mm i.d., 100 mm long, 3 μm particles (Waters) | | | | |
|  | Column temperature | 40 °C | | | | |
|  | Mobile phase | A: 0.1% formic acid and 10 mM ammonium acetate in water | | | | |
|  |  | B: Acetonitrile | | | | |
|  | Mobile phase gradient | 0–4 min | A95 | B5 |  |  |
|  |  | 4–15 min | A95>50 | B5>50 |  |  |
|  |  | 15–18 min | A50>0 | B50>100 |  |  |
|  |  | 18–23 min | A0 | B100 |  |  |
|  |  | 23–30 min | A90 | B10 |  |  |
|  | Mobile phase flow | 0.2 mL min^−1^ | | | | |
|  | Injection volume | 10 μL | | | | |
|  |  |  |  |  |  |  |
| Mass spectrometer | |  | | | | |
|  | Instrument | TripleQuad 6500 (AB SCIEX) | |  |  |  |
|  | Ionization | Electrospray positive ionization (multiple reaction monitoring) | | |  |  |
|  | Monitored ion (m/z) | Quantification | Confirmation | Declustering potential (V) | Collision energy (V) | Retention time (min) |
|  | Acetamiprid | 223.0 > 126.0 | 223.0 > 90.0 | 71 | 29 | 12.7 |
|  | Clothianidin | 249.9 > 169.0 | 249.9 > 132.0 | 21 | 19 | 11.7 |
|  | Dinotefuran | 203.0 > 129.0 | 203.0 > 114.1 | 1 | 17 | 8.4 |
|  | Imidacloprid | 256.0 > 175.1 | 256.0 > 209.0 | 56 | 25 | 12.1 |
|  | Nitenpyram | 271.0 > 99.0 | 271.0 > 125.9 | 51 | 39 | 9.7 |
|  | Thiacloprid | 252.9 > 125.9 | 252.9 > 90.0 | 76 | 29 | 13.9 |
|  | Thiamethoxam | 291.8 > 211.1 | 291.8 > 181.0 | 41 | 17 | 10.7 |
|  | Desmethyl-acetamiprid | 209.1 > 125.9 | 209.1 > 90.0 | 61 | 25 | 11.9 |
|  | Desmethyl-thiamethoxam | 278.0 > 132.0 | 278.0 > 174.0 | 36 | 25 | 12.6 |
|  | Thiacloprid amide | 271.0 > 125.9 | 271.0 > 73.0 | 31 | 35 | 11.8 |
|  | Acetamiprid-d3 | 226.0 > 126.0 |  | 71 | 31 | 12.7 |
|  | Acetamiprid-d6 | 226.0 > 126.0 |  | 71 | 31 | 12.7 |
|  | Clothianidin-d3 | 253.0 > 172.1 |  | 1 | 19 | 11.7 |
|  | Dinotefuran-d3 | 206.1 > 132.1 |  | 56 | 19 | 8.4 |
|  | Imidacloprid-d4 | 260.1 > 179.1 |  | 26 | 25 | 12.1 |
|  | Thiacloprid-d4 | 296.0 > 215.0 |  | 91 | 29 | 13.9 |
|  | Thiamethoxam-d4 | 296.0 > 215.0 |  | 41 | 17 | 10.7 |
|  | Desmethyl-acetamiprid-d3 | 212.1 > 125.9 |  | 61 | 25 | 11.9 |
