# Supplementary material for: Biological Monitoring of Human Exposure to Neonicotinoids Using Urine Samples, and Neonicotinoid Excretion Kinetics
Source: PLoS One. 2016 Jan 5;11(1):e0146335. doi: 10.1371/journal.pone.0146335 (PMC4701477; doi:10.1371/journal.pone.0146335)
Supplement: S3 Table — (DOCX) [file pone.0146335.s006.docx]

| **S3 Table.** Calibration curves, detection limits, and recoveries for the neonicotinoids | | | | | | |
| --- | --- | --- | --- | --- | --- | --- |
|  | Range | Calibration curves (Y=ax+b)^a^ | | | Detection limit ^b^ | Recovery  % (RSD%)^c^ |
| Target analytes | (ng mL^−1^) | Slope (a) | Intercept (b) | Linearity (r) | (ng mL^−1^) | (n=8) |
| Acetamiprid | 0.005–2 | 0.886 | 0.00529 | 0.9999 | 0.005 | 91 (10) |
| Clothianidin | 0.02-5 | 0.905 | −0.00721 | 0.9999 | 0.020 | 100 (15) |
| Dinotefuran | 0.01–10 | 1.29 | 0.00201 | 0.9999 | 0.010 | 64 (21) |
| Imidacloprid | 0.01–1 | 0.881 | 0.0168 | 0.9999 | 0.010 | 97 (17) |
| Nitenpyram | 0.01–2 | 7.87E+05 | 1.04E+03 | 0.9999 | 0.010 | 72 (6) |
| Thiacloprid | 0.005–2 | 1.02 | 0.00442 | 0.9999 | 0.005 | 80 (5) |
| Thiamethoxam | 0.01–1 | 0.909 | 0.00396 | 0.9999 | 0.010 | 89 (16) |
| Desmethyl-acetamiprid | 0.005–2 | 1.66E+06 | 8.55E+03 | 0.9999 | 0.005 | 72 (12) |
| Desmethyl-thiamethoxam | 0.02–2 | 2.43E+05 | 2.30E+03 | 0.9999 | 0.020 | 75 (4) |
| Thiacloprid amide | 0.005–2 | 4.24E+06 | 1.30E+04 | 0.9999 | 0.005 | 69 (8) |
|  |  |  |  |  |  |  |
| ^a^ Y and X are the area and concentration (ng/mL), respectively, for the external standard method and the analyte area / internal standard area ratio and analyte concentration / internal standard concentration ratio, respectively, for the internal standard method. | | | | | | |
| ^b^ 1 mL sample |  |  |  |  |  |  |
| ^c^ RSD: relative standard deviation | | | | | | |
