# Supplementary material for: Biological Monitoring of Human Exposure to Neonicotinoids Using Urine Samples, and Neonicotinoid Excretion Kinetics
Source: PLoS One. 2016 Jan 5;11(1):e0146335. doi: 10.1371/journal.pone.0146335 (PMC4701477; doi:10.1371/journal.pone.0146335)
Supplement: S4 Table — (DOCX) [file pone.0146335.s007.docx]

| **S4 Table.** Amounts of neonicotinoids excreted in urine over 96 h after a 5 μg microdose of each deuterium-labeled compound was ingested, and the percentage of the initial parent compound dose that was recovered | | |
| --- | --- | --- |
|  | Recovery in urine (μg) |  |
|  | mean±SD | % |
| Acetamiprid-d6 | 0.13±0.17 | 2.6±3.4 |
| Clothianidin-d3 | 3.18±0.73 | 63.7±14.6 |
| Dinotefuran-d3 | 4.64±1.11 | 92.8±22.1 |
| Imidacloprid-d4 | 0.64±0.35 | 12.7±7.0 |
| Desmethyl-acetamiprid-d3 | 1.53±0.78 | 30.7±15.6 |
