# Supplementary material for: Biological Monitoring of Human Exposure to Neonicotinoids Using Urine Samples, and Neonicotinoid Excretion Kinetics
Source: PLoS One. 2016 Jan 5;11(1):e0146335. doi: 10.1371/journal.pone.0146335 (PMC4701477; doi:10.1371/journal.pone.0146335)
Supplement: S5 Table — (DOCX) [file pone.0146335.s008.docx]

**S5 Table.** Estimated daily intakes of neonicotinoids found in the microdose study

|  | *M* (μg day^−1^) |  | *N* | *R*^2^ |
| --- | --- | --- | --- | --- |
| Clothianidin | 1.26 ± 1.12 |  | 90 | 0.20 (*p* << 0.05) |
| Imidacloprid | 1.58 ± 3.37 |  | 89 | 0.11 (*p* = 0.0012) |
| Dinotefuran | 5.18 ± 6.40 |  | 83 | 0.077 (*p* = 0.011) |
| Desmethyl-acetamiprid | 2.93 ± 12.4 |  | 93 | 0.057 (*p* = 0.021) |

*M* (Mean ± SD) was estimated using Eqs. 13, 15, and 18 (see Supporting Information, methods), assuming the steady state conditions occurred before the single 2 μg dose was ingested. The *R*^2^ values are the correlation coefficients for the relationships between the observed and modeled amounts excreted.
